# Excimer laser in contrast for the treatment of acute MI caused by thrombosis of underexpanded stent

**DOI:** 10.1002/ccr3.1537

**Published:** 2018-06-13

**Authors:** Zaheed Tai

**Affiliations:** ^1^ Bostick Heart Center Winter Haven Hospital Winter Haven Florida

**Keywords:** Atherectomy, atherotomy, excimer laser, myocardial infarction, thrombosis

## Abstract

The use of excimer laser with contrast for treating an underexpanded stent in the setting of subacute stent thrombosis and hemodynamic instability is described. The patients presented with acute coronary syndromes and cardiogenic shock resulting from stent thrombosis of underexpanded stents. The stents were recalcitrant to aggressive balloon dilation and in the setting of an acute myocardial infarction; rotational atherectomy is a relative contraindication. The use of concurrent contrast during laser atherectomy resulted in plaque modification and subsequent stent expansion.

## Introduction

In the current drug‐eluting stent (DES) era, early or definite stent thrombosis has been reported to occur in approximately 1.4% of the cases 1, 2. Several factors can contribute to stent thrombosis but can typically be categorized into three broad categories: (1) patient‐related factors (tobacco, diabetes, and renal insufficiency), (2) lesion‐related factors (thrombotic lesion, diffuse disease) and (3) stent‐related factors. Stent‐related factors include stent underexpansion, edge dissection, delayed stent endothelialization, strut fracture, stent malapposition, neoatherosclerosis, and hypersensitivity to the stent polymer. Stent underexpansion is usually the result of inadequate vessel preparation or unrecognized calcium. Moderate‐to‐severe calcium is present approximately 30% of the time 3. These lesions can be difficult to treat or resistant to conventional therapy resulting in underexpanded stents. The typical approach in this scenario is aggressive balloon dilation with noncompliant balloons. However, if this is not successful, alternative solutions must be considered such as atherectomy or surgery.

The use of excimer laser coronary angioplasty (ELCA) to treat resistant lesions and underexpanded stents has been previously reported 4, 5, 6. These cases were elective cases in either newly deployed or previously endothelialized stents. Veerasamy et al. reported two cases of laser use in the setting of ST elevation, but there was no report of hemodynamic instability 7. Two cases of laser energy application to treat patients in the setting on an acute myocardial infarction with cardiogenic shock secondary to stent thrombosis of underexpanded stents are presented. In both cases, excimer laser with contrast was used to modify the plaque under the struts to facilitate optimal stent expansion and restore normal flow in the coronaries. This appears to be the first reported cases utilizing this technique in this clinical setting.

## Case 1

**Figure 1:**
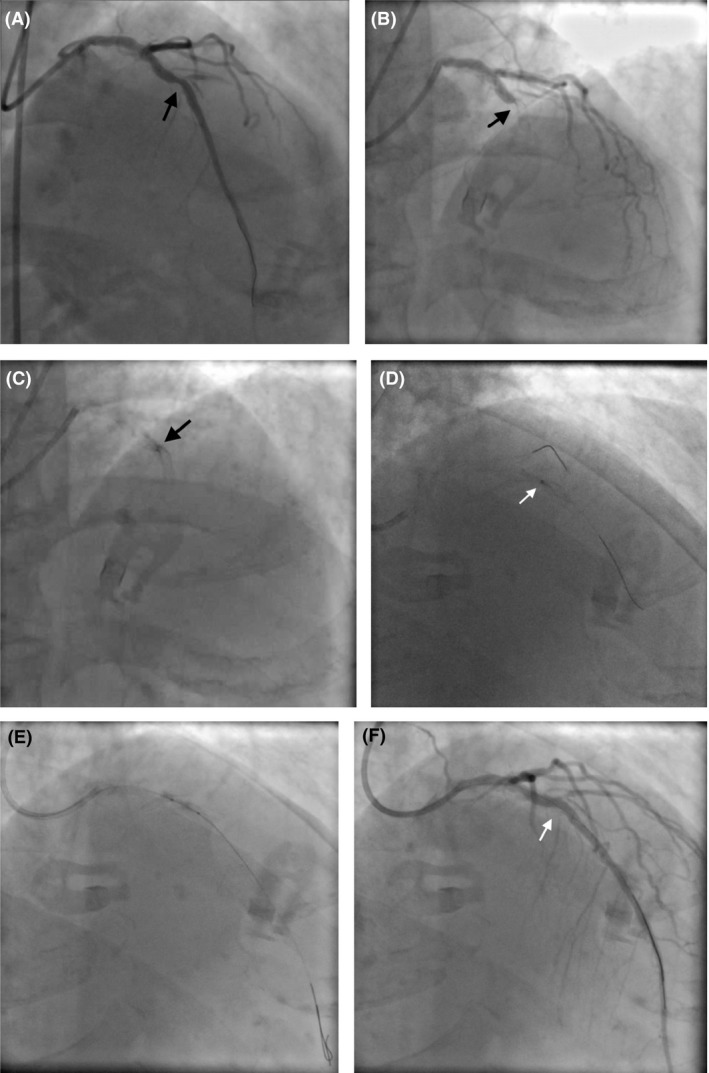
(A) final angiogram from initial procedure with underexpanded stent; (B) angiogram on presentation demonstrating occlusion at site of underexpanded stent; (C) underexpanded stent at overlap; (D) 0.9 ELCA; (E) NC balloon; (F) final angiogram with expansion of stent.

A 79‐year‐old male with a history of hypertension, hyperlipidemia, and polycythemia vera underwent PCI of the left anterior descending artery (LAD) 1 week prior to admission at an outside institution with overlapping 3.0 × 15 mm Integrity bare‐metal stents (Medtronic) to the mid‐LAD (Fig. 1A). He developed chest pain approximately 40 min prior to arrival in the ER where he was found to have anterior ST elevations with hemodynamics consistent with shock. He was taken emergently to the laboratory where angiography with a 6 Fr system was performed via the right radial approach demonstrated a 100% occlusion of the LAD with in‐stent thrombosis (Fig. 1B). There was also angiographic underexpansion of the stents at the site of occlusion (Fig. 1C). In the interim from arrival, we were able to obtain his catheterization report from the other hospital, and it was reported that an attempt was made to postdilate the stents with a 3.0 and 3.25 noncompliant (NC) balloon without resolution of the “waste” in the midportion of the stent. Bivalirudin was administered, and a Runthrough wire (Terumo) was passed distally restoring antegrade TIMI 1 flow. We then advanced a 0.9 laser to the lesion (Fig. 1D). The catheter would not advance through the lesion. Given the recent issues encountered by the previous operator, we performed laser with contrast injections at a setting of 80/80 (fluency/rate) for approximately 1 min. We then did aggressive dilation with a 2.5 and then a 3.0 NC balloon with expansion of the stent (Fig. 1E). He stabilized hemodynamically at this point. We then performed intravascular ultrasound (IVUS) demonstrating severe concentric calcification and lumen diameter of about 3.75–4.0 mm. Postdilation was performed with a 4.0 × 12 Quantum balloon (Boston Scientific) with an excellent angiographic result (Fig. 1F). Final IVUS demonstrated good stent expansion; however, on pullback, there was evidence of significant distal left main and ostial LAD disease with heavy calcification; this was also appreciated in the LAO cranial views. After the patient was stabilized and recovered from his event (echo demonstrated a decline in the ejection fraction (EF) to approximately 25% with anterior wall motion abnormality compared with a previously reported normal EF), a heart team approach was used to determine the best revascularization option for the patient. Given the extent of calcification and anatomic concerns, he eventually underwent bypass and was discharged home on postoperative day five.

## Case 2

**Figure 2:**
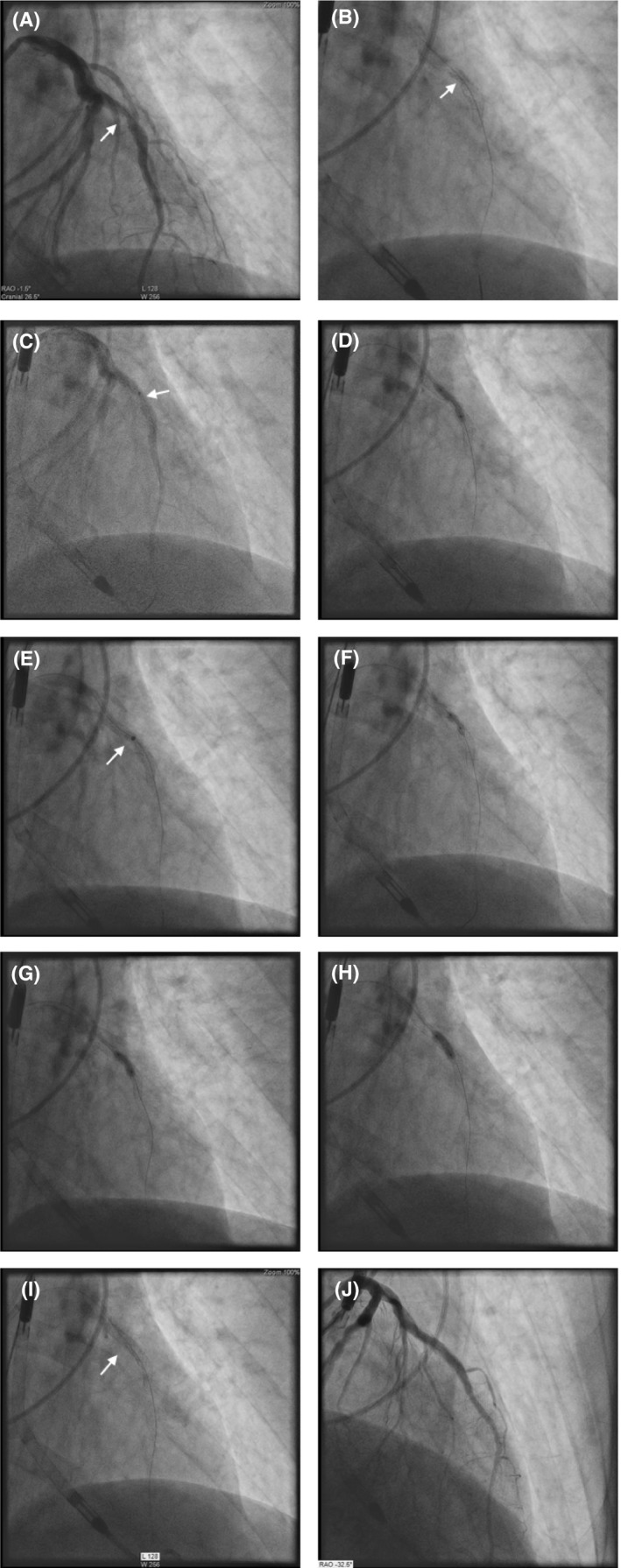
(A) initial angiogram with TIMI 1 flow and underexpanded stent; (B) underexpanded stent at overlap; (C) 0.9 ELCA with contrast; (D) dilation with NC balloon at high pressure after 0.9 ELCA demonstrates failure to expand; (E) 1.4 ELCA with contrast, catheter did not cross lesion; (F) NC balloon at high pressure post‐ELCA with “waste”; (G) Flextome at 14 atm; (H) Flextome at 24 atm; (I) expansion of stent; (J) final angiogram with TIMI 3 flow and expanded stent.

A 73‐year‐old male with history of hypertension, hyperlipidemia, and esophageal reflux presented to an outside hospital with a non‐ST elevation myocardial infarction and underwent PCI of the LAD with overlapping 3.0 × 38 and 3.5 × 18 Resolute DES (Medtronic). There was a suboptimal stent expansion in the LAD, and he was discharged on dual antiplatelet (DAPT) therapy. He presented to the same institution 6 days later with acute ST elevation and stent thrombosis. They were able to pass a wire and did sequential balloon inflations sizing up to a 4.0 balloon. Antegrade flow was restored, but despite aggressive dilation, the lesion did not expand. He was transferred to our institution for bypass. The surgical team evaluated him, and it was felt PCI would be a better option if feasible rather than surgery in the acute situation. In the interim, the patient developed cardiogenic shock and chest pain and was taken emergently to the laboratory where an Impella CP (Abiomed) was placed to stabilize the patient. A decision was made to perform laser atherectomy of the stent to try and expand the stent. Right radial access was obtained with a 6/7 slender sheath (Terumo). Angiography revealed a patent LAD with TIMI 1 flow (Fig. 2A) and underexpansion of the stents at the overlap (Fig. 2B). A 0.9 ELCA catheter was easily advanced through the lesion (Fig. 2C). Laser atherectomy was performed with contrast at 80/80 (fluency and rate) for 2 min and then an attempt to dilate with a series of 2.5 and 3.0 NC balloons was performed. Despite high‐pressure inflation at 26 atm, the lesion did not yield (Fig. 2D). Perhaps rupturing the balloon would have resulted in some plaque modification as well; however, this did not occur. It was felt that the 0.9 ELCA did not have enough contact with the underlying plaque given the preexisting channel (although underexpanded). Therefore, a 1.4 ELCA catheter was advanced to the lesion; however, it would not advance through the lesion. Atherectomy was performed at 60/40 for 1 min with contrast puffs during atherectomy (Fig. 2E) followed by attempted dilation with a 3.0 NC balloon without resolution of the lesion (Fig. 2F). It was decided to use an atherotomy balloon at this time to increase the radial force. A 3.0 × 10 Flextome was advanced with an initial inflation at 14 atm (Fig. 2G) without resolution. The lesion yielded at 24 atm (Fig. 2H). Postdilation was then performed with a 3.75 NC balloon with restoration of TIMI 3 flow (Fig. 2I and J). The patient was weaned off the Impella and had no acute issues postprocedure.

## Discussion

The Food and Drug Administration approved laser atherectomy in 1992 for its first clinical use, since then it has been used as adjunctive therapy to treat a variety of coronary lesion subsets. Early success was tempered by concerns of laser‐induced complications, in particular dissection and perforation. Refinement in technique, in particular saline flush and equipment (pulsed lasers instead of continuous wave lasers and low thermal effect “cool” lasers), resulted in better outcomes. Although atherectomy has not been demonstrated to be superior to conventional angioplasty 8, there are certain lesion subsets where it has a role to facilitate stent delivery and allow for better stent expansion. Current indications for laser atherectomy include total occlusions crossable by a guidewire, in‐stent restenosis prior to brachytherapy, long lesions, ostial lesions, saphenous vein grafts, and previously failed angioplasty 8, 9. The current ELCA catheter consists of a flexible fiber‐optic catheter connected to an external laser energy source. The machine emits laser with an output flow between 30 and 80 mJ/mm^2^ (fluence) and a repetition rate of 25–80 pulses/sec (rate). The catheter is available in a range of sizes: 0.9, 1.4, 1.7, and 2.0 mm. The tip of the catheter emits pulses of laser light, which modifies various plaque (fibrous tissue, calcium, soft atheroma, and thrombus) by three mechanisms: photochemical (breaking molecular bonds), photothermal (turning intracellular water to vapor), and photomechanical (creating kinetic energy through expansion and collapse of the vapor bubble) effects. Complications from early laser atherectomy usually were the result of two processes 10:

1. Absorption of laser energy by blood and contrast resulting in a rapidly expanding and contracting vapor bubble causing dilation and invagination of the arterial wall 11, 12.
2. Increase in vessel wall and plaque temperature by exceeding the thermal relaxation time of the irradiated tissue 13, 14.

These issues have been mitigated in commercially available devices by the use of the pulsed excimer laser and saline flush prior to activation of the laser to flush contrast and blood from the vessel.

In both the cases presented, complete stent expansion was not achieved initially despite high‐pressure balloon inflation with noncompliant balloons that were angiographically sized 1:1 or larger to the reference vessel. This was likely due to coronary calcification and resilience of the plaque. Remedies to treat underexpanded stents include aggressive balloon dilation, rotational atherectomy, or laser atherectomy. Aggressive dilation has the potential to result in coronary perforation, dissection, or balloon rupture with a high‐pressure contrast jet causing subintimal dissection. However, this method is readily available and does not require any additional capital equipment; hence, it is typically the first approach to treat underexpanded stents. This can be modified with the use of a buddy wire or atherotomy balloon. The “focal force” created may reduce circumferential plaque dissection and enhance longitudinal plaque fissuring. This should ideally be performed prior to stent implant to provide maximum contact with the plaque surface. The atherotomy balloon's blade (Flextome) or scoring element (AngioSculpt) cannot reach the plaque once the stent is implanted. When a lesion proves resistant to this approach, alternative options need to be considered which can include atherectomy or surgical revascularization. Surgery has inherited risk and may not be ideal in the acute setting especially if there is hemodynamic compromise.

High‐speed rotational atherectomy (HSRA) has been described in case reports to be effective in treating underexpanded stent; however, this approach is relatively contraindicated in thrombus containing lesion because of the potential for distal embolization or no reflow 15. This concern may be further exacerbated in the presence of cardiogenic shock. Additional concerns of HSRA in stents include strut embolization, dissections, and burr entrapment 16, 17, 18. Rotational atherectomy may eventually contact the underlying plaque but inevitably results in partial removal of the stent strut and potential for vessel damage. Ferri et al. 19 recently reported a case series of 16 patients in which they effectively used rotational atherectomy to treat underexpanded stents with acceptable results. Procedural success was less than observed when compared to the use of laser atherectomy in the ELLEMENT registry 20 and associated with high rate of target lesion revascularization 16. The ELLEMENT registry reported the use of laser atherectomy with contrast injection to treat underexpanded stents in 28 patients (25 with ISR and three with underexpanded stent in de novo lesions) and excluded patients with an acute MI.

Laser atherectomy with and without contrast has previously been used to expand underexpanded stents 6, 7, 8, 20, 21. The absorption of laser energy by tissue leads to ablation by photochemical, photomechanical, and photothermal effects. In the presence of contrast, the predominant effect is a thermomechanical action of the rapidly expanding and imploding vapor bubble. This rapid process generates pressure waves that propagate away from the irradiated tissue (acousticomechanical effect). This results in the beneficial as well as detrimental effects of laser atherectomy. Tcheng et al. 22 demonstrated that no pressure waveform is generated with saline while increased pulse pressure is generated with exposure to blood and magnified more with exposure to contrast. Hence the “flush and bathe” technique was utilized to minimize the risk of utilizing laser atherectomy. Goldberg et al. 21 were the first to describe the use of contrast injection to amplify the effects of laser atherectomy to successfully expand a stent refractory to balloon dilation. Other case studies have demonstrated the use of laser with contrast to facilitate expansion of an underexpanded stent 5, 6, 7, 20 in stable patients. It appears this is the first reported use of this technique to treat underexpanded stents in the setting of an acute myocardial infarction with hemodynamic instability. Optical coherence tomography (OCT) imaging has demonstrated that saline infusion with laser results in only minor dissection and intimal erosion, where as in the presence of contrast, the laser disrupted the calcified plaque behind the stent 23. The presence of contrast can increase the pressure pulses to >100 atmospheres and disrupt the plaque underneath the struts even without direct contact with the plaque. This approach can increase the potential for cavitation and dissections resulting from laser atherectomy; however, the presence of the stent may minimize the risk of vessel injury. Adjunctive therapy with an atherotomy balloon (or a “focal force: technique), in this case a cutting balloon, provides better torsional, longitudinal, and radial force compared with balloon angioplasty. The microtome edge typically initiates an indentation into the plaque, after which the shear force applied by the balloon inflation propagates the crack. Although there was the presence of struts in these cases, the enhanced radial force of the balloon was the rationale for its use. Depending on balloon size, the cutting force at the blade edge is enhanced 200,000–400,000 times 24.

In addition to treating the underexpanded stent, the use of laser atherectomy in the setting of acute MI has additional potential clinical benefits. The excimer laser has demonstrated utility in thrombus laden lesion and can ablate thrombus as well as underlying plaque, inhibit platelet aggregation, and separate thrombi from the arterial walls. Angioplasty alone can result in thrombus displacement causing embolization and platelet aggregation. Thrombectomy alone may remove thrombus but does not affect underlying atherosclerotic plaque. Laser atherectomy ablates thrombus, modifies the underlying atherosclerotic plaque, and can restore antegrade flow with the potential to minimize no reflow. In addition, this may enhance IIb/IIIa activity or facilitate adjunctive thrombectomy 25, 26.

This technique should be used with some caution. First, consider starting with a 0.9 excimer laser; the catheter is more deliverable than larger catheters, and the vapor bubbles generated can be two to three times the diameter of the catheter 27. This effect can be amplified in the presence of blood or contrast. Large catheters carry the potential for slow flow as a result of macro bubbles. Typically the catheter is sized to two‐third the vessel size, but the usual workhorse catheter in the coronaries is the 0.9 and occasionally the 1.4. Unlike the rotablator, the laser catheter does not necessarily need to cross the lesion or come in direct contact to cause plaque modification. Second, avoid tortuous vessels and monitor wire bias. Atherectomy in this setting can result in vessel perforation or dissection. Third, choose your guiding catheter size based on the size of the laser to be used and try to avoid the use of side holes. Although a 1.4 catheter will fit in a 6 Fr guide, you may not get adequate enough contrast “puffs” in a 6 Fr system. That is why we chose a 7 Fr system for the second case. This allowed the contrast to pass around the catheter during the procedure. Fourth, consider using this approach in a 6 Fr or larger system. Given the potential risk of perforation with this technique, a 6 Fr or larger system will allow use of GraftMaster (Abbott) stent if warranted.

## Conclusion

This approach is a niche approach for a fortunately rare but complex lesion. It is an off‐label use of the product. Previously reported cases have demonstrated relative safety, but there is the potential for complications. However it appears to be better when compared to rotational atherectomy 19, 20. In the current cases, the excimer laser served two purposes, ablating thrombus and underlying plaque and facilitating previously failed stent expansion by plaque modification. Another limitation of this technique is the lack of availability of the ELCA; however, as more operators are tackling complex lesions, this device may become a part of their toolbox.

## Authorship

ZT: are author and operator for cases.

## Conflict of Interest

None declared.
